# Predicting Carcinogenic Mechanisms of Non-Genotoxic Carcinogens via Combined Analysis of Global DNA Methylation and In Vitro Cell Transformation

**DOI:** 10.3390/ijms21155387

**Published:** 2020-07-29

**Authors:** Sung-Hee Hwang, Hojin Yeom, Byeal-I Han, Byung-Joo Ham, Yong-Moon Lee, Mi-Ryung Han, Michael Lee

**Affiliations:** 1 Division of Life Sciences, College of Life Sciences and Bioengineering, Incheon National University, Incheon 22012, Korea; sunghee7518@naver.com (S.-H.H.); ty010@naver.com (H.Y.); 2 Institute for New Drug Development, Incheon National University, Incheon 22012, Korea; tmxkgks1@naver.com; 3 Department of Psychiatry, Korea University Anam Hospital, Korea University College of Medicine, Seoul 02841, Korea; hambj@korea.ac.kr; 4 College of Pharmacy and Medical Research Center, Chungbuk National University, Cheoungju-si, Chungcheongbuk-do 28160, Korea; ymleefn@gmail.com; 5 INU Human Genome Center, Incheon National University, Incheon 22012, Korea

**Keywords:** non-genotoxic carcinogen, in vitro cell transformation assay, methylation profiles, RRBS

## Abstract

An in vitro cell transformation assay (CTA) is useful for the detection of non-genotoxic carcinogens (NGTXCs); however, it does not provide information on their modes of action. In this study, to pursue a mechanism-based approach in the risk assessment of NGTXCs, we aimed to develop an integrated strategy comprising an in vitro Bhas 42 CTA and global DNA methylation analysis. For this purpose, 10 NGTXCs, which were also predicted to be negative through Derek/Sarah structure–activity relationship analysis, were first tested for transforming activity in Bhas 42 cells. Methylation profiles using reduced representation bisulfite sequencing were generated for seven NGTXCs that were positive in CTAs. In general, the differentially methylated regions (DMRs) within promoter regions showed slightly more bias toward hypermethylation than the DMRs across the whole genome. We also identified 13 genes associated with overlapping DMRs within the promoter regions in four NGTXCs, of which seven were hypermethylated and six were hypomethylated. Using ingenuity pathway analysis, the genes with DMRs at the CpG sites were found to be enriched in cancer-related categories, including “cell-to-cell signaling and interaction” as well as “cell death and survival”. Moreover, the networks related to “cell death and survival”, which were considered to be associated with carcinogenesis, were identified in six NGTXCs. These results suggest that epigenetic changes supporting cell transformation processes occur during non-genotoxic carcinogenesis. Taken together, our combined system can become an attractive component for an integrated approach for the testing and assessment of NGTXCs.

## 1. Introduction

Non-genotoxic carcinogens (NGTXCs) induce tumors through a variety of non-DNA-damaging mechanisms during carcinogenesis. Twelve percent (45/371) of IARC Group 1, 2A, and 2B carcinogens appear to be non-genotoxic [1]. NGTXCs are traditionally identified only in vivo in two-year rodent bioassays because there are currently no accurate or well validated in vitro assays. In vitro cell transformation assays (CTAs), which mimic different stages of the in vivo neoplastic process, have been reported to be useful for detecting possible NGTXCs [2,3]. In particular, CTAs using Bhas 42 cells are capable of detecting promotor activities without initiator treatment, and thus are superior to those of the BALB/c 3T3 CTA and other CTAs (Validation Study Report [4,5]). In recent years, the Bhas 42 CTA has undergone several comprehensive international validation studies [4,6,7] and is now the subject of an OECD guidance document [8].

The importance of a mechanism-based approach in the risk assessment of NGTXCs has risen. However, current CTAs do not provide information on key molecular events supporting the carcinogenesis process of NGTXCs. In fact, NGTXCs have a number of relatively diverse modes of action, including epigenetic alterations, tumor promotion, endocrine modulation, immune suppression, and inflammatory responses [1,9,10,11]. Owing to this diversity, it is impossible to detect all NGTXCs in one test system [1]. Presumably, the performance of a test battery to cover all the modes of action of NGTXCs is time-consuming and complex. Many studies have highlighted that the mode of action of NGTXCs may involve epigenetic alteration, including altered DNA methylation, histone modification, non-coding RNA, and chromatin remodeling [12,13]. With this, it has become clear that epigenetic dysregulation is a characteristic of virtually all human cancers [14]. Of these alterations, DNA methylation is demonstrably heritable [15], rendering DNA methylation the standard epigenetic marker [16]. Furthermore, changes in gene expression patterns based on altered DNA methylation have been linked to various cancers [17]. Using a massively parallel sequencing-based approach, it is relatively easy to generate comprehensive genome-wide methylation profiles. Reduced-representation bisulfite sequencing (RRBS) is an economical, accurate, and efficient method for analyzing the DNA methylation of a single base [18,19]. This method enriches the promoter and CpG island regions by methylation-insensitive restriction endonuclease digestion, providing a locus-specific methylation analysis for roughly 6–8% of the CpG loci in the human genome [20]. Moreover, several laboratories have combined in vitro CTAs with several endpoint applications, such as protein analysis [21] or whole-genome transcriptomic profiles [22,23], to elucidate cancer mechanisms in more detail. However, little has been reported on an integrated strategy consisting of the methylation analysis and in vitro CTA. We previously reported a method that couples the in vitro Bhas 42 CTA with methyl-CpG-binding domain protein sequencing (MBD-Seq) to utilize the CTA within an integrated approach for testing and assessment (IATA) for NGTXCs [24].

Herein, we analyzed the methylation profiles of transformed foci induced by seven NGTXCs using the RRBS method to distinguish the different modes of action of NGTXCs with the aim of predicting essential information regarding their carcinogenic mechanisms. Combining restriction enzymes and bisulfite sequencing, RRBS enriches genomic regions with a high CpG content [25]. Furthermore, RRBS can be applied to quickly identify aberrant methylation in cancer because this technique is highly sensitive [26]. As such, we provide information regarding the methylation signatures in foci during NGTXC-induced cell transformation and reveal, using ingenuity pathway analysis (IPA), that the genes with differentially methylated regions (DMRs) at CpG sites are linked to cancer-related categories in the enriched functional annotations. As far as we know, this study is among the first to elucidate a unique pathway associated with NGTXC-induced carcinogenesis based on a genome-wide methylation approach.

## 2. Results

### 2.1. Selection of Test Articles

The selection of 10 NGTXCs was based on carcinogenicity and genotoxicity reviews by previously published reports [5,27,28] (Table 1). The selected 10 NGTXCs were also predicted to be negative through Derek/Sarah analysis, which is accepted by regulators under International Conference on Harmonisation (ICH) guideline M7 on the assessment and control of DNA-reactive impurities in pharmaceuticals [29]. In particular, a structural alert for non-genotoxic carcinogenicity was noted in four NGTXCs (DEHP, TCDD, rosuvastatin, and sodium saccharin) by the OECD quantitative structure–activity relationship (QSAR) toolbox [30,31]. Table 2 lists the diversity in the modes of action of 10 NGTXCs. The non-genotoxic modes of action include the dysregulation of the hepatic iron metabolism, choline deficiency, peroxisome proliferation, and calculus formation.

### 2.2. Transformation Assay

As a first step towards the development of an integrated approach, the in vitro Bhas 42 CTA suggested by the OECD Working Group [8] was performed on 10 NGTXCs. Cell growth assays were conducted to identify the most suitable concentration of each test article for the Bhas 42 CTA (data not shown). Three independent assays were carried out to assess cytotoxicity. Five to seven concentrations of each NGTXC were established based on the results of the cell growth assay. The highest concentration for the cell growth assay was set to 10 mM or 2 mg/mL according to an OECD guidance document [8]. In the case of insoluble substances, the concentration range for the growth assay was set to include one concentration with a visible precipitate due to the limit of solubility. However, in the transformation assay for melamine, rosuvastatin, and D-limonene, no foci were formed even in the concentration range where no cytotoxicity was observed in the cell growth assay (data not shown). Thus, for these three test substances, the CTA was performed at a re-adjusted concentration range, which is low enough to form foci. The promoter activity of 10 NGTXCs was examined, and the results are summarized in Table 3. Among the 10 NGTXCs, three (D-limonene, melamine, and rosuvastatin) showed negative results in the promotion test. Sodium saccharin, methapyrilene, DEHP, diethanolamine (DEA), TCDD, and cholic acid induced a more than two-fold increase in foci formation, while okadaic acid led to a relatively weak increase.

### 2.3. Identification and Distribution of DMRs

**Figure 1:**
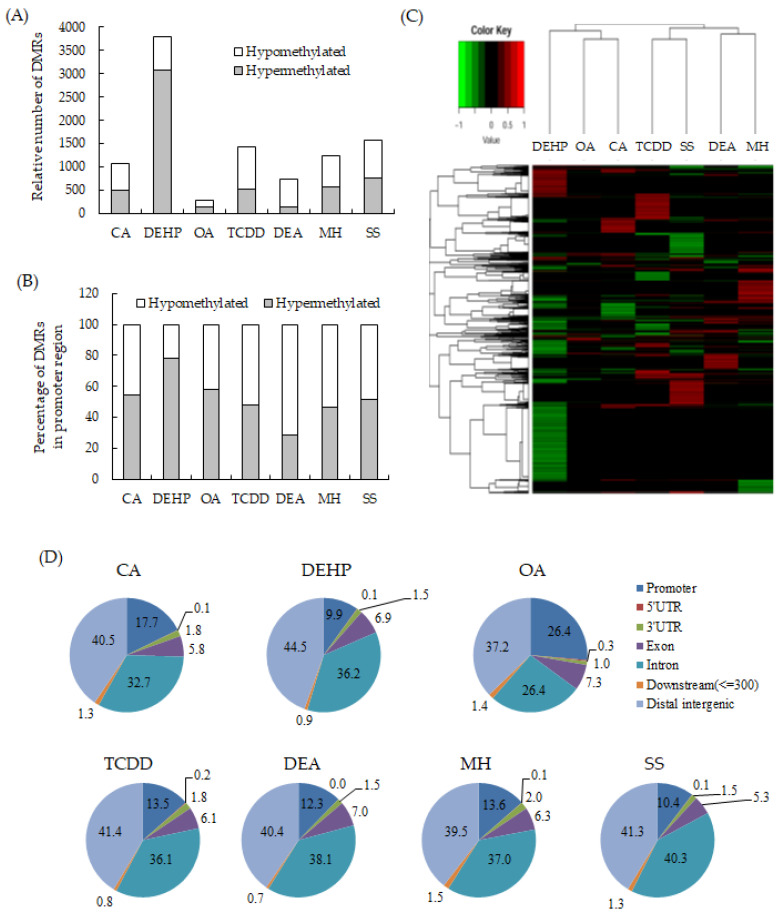
Identification and distribution of the differentially methylated regions (DMRs) specific to each non-genotoxic carcinogen (NGTXC) during the cell transformation. (**A**) Histogram of the distribution of the relative number of hypermethylated and hypomethylated DMRs per NGTXC. (**B**) Histogram of DMRs in the promoter region (−1500–1500). In (**A**,**B**), the DMRs that were unique to each NGTXC-induced transformed focus were separated by methylation status and classified as hypermethylated or hypomethylated with respect to the untransformed cells. (**C**) The heatmap of the DNA methylation level at DMRs specific to each NGTXC. Each column represents a different NGTXC, and each row represents a different DMR specific to each NGTXC. The red color represents hypermethylation, while the green color represents hypomethylation. (**D**) The distribution of DMRs in different genomic regions: 3′-UTR, 5′-UTR, exon, intron, intergenic, promoter, and downstream (≤300).

To detect the critical methylation events associated with NGTXCs, we compared the DNA methylomes from seven Bhas 42 CTA-positive NGTXCs via RRBS analysis, which is an effective and economical method for analyzing the DNA methylation of a single base [18,19]. DMRs, defined as genomic regions exhibiting methylation differences with more than 20% changes between the samples (untransformed cells vs. transformed foci) at a CpG site and achieving a false discovery rate (FDR)-adjusted *p*-value < 0.05, were used for all the subsequent analyses. The complete list of DMRs associated with each NGTXC is included in Supplementary Tables S1–S7. The average number of DMRs identified in each NGTXC are 730–1574, with the exception of 3796 DMRs for DEHP and 288 DMRs for okadaic acid (OA). DMRs identified in OA were relatively low compared to other CTA-positive NGTXCs probably due to the low transforming activity of OA as shown in Table 3. We calculated the coefficient of correlation to investigate whether there was any relation between the number of DMRs and in vitro cell transformation. The number of DMRs appeared to be positively related to the number of foci formed in CTA, but the coefficient of correlation was low with the value of 0.4782. The scatter diagram and coefficient of correlation are presented in Supplementary Figure S1. Interestingly, of the 3796 DMRs in DEHP, 3081 DMRs (81%) were hypermethylated, while the remaining 715 (19%) were hypomethylated (Figure 1A). Conversely, in DEA-induced transformed foci, 730 significant DMRs were detected, of which 146 (20%) were hypermethylated and 583 (80%) were hypomethylated. The DMRs in the promoter region, which were defined as DMRs within 1500 bp upstream and 1500 bp downstream from the transcription start site (TSS), were then segregated on the basis of methylation status (Figure 1B). With the exception of DEHP, the DMRs in the promoter regions showed slightly more bias toward hypermethylation than DMRs throughout the whole genome. Figure 1C shows the heatmap of the DNA methylation level at DMRs specific for each NGTXC using hierarchical agglomerative clustering with complete linkage.

Then, the distributions of the DMRs from the RRBS data were compared in terms of the following seven genomic features: (i) promoters (DMR within 1500 bp upstream and 1500 bp downstream from TSS); (ii) exon; (iii) intron; (iv) 5′-UTR; (v) 3′-UTR; (vi) downstream (a 3-kb region downstream of transcription termination sites); and (vii) distal intergenic regions. The distribution patterns appeared to be relatively similar among the six NGTXCs, except for OA (Figure 1D). Almost 40% of the identified DMRs were associated with the gene body (exon + intron). DMRs located within the intergenic regions were almost 40%. We also discovered that, on average, 15% of the identified DMRs were located in promoter regions, which are crucial for gene expression regulation. However, in the case of OA, DMRs were observed in the promoter regions at a frequency of 26.4%.

### 2.4. Overlapping DMRs in Promoter Regions across NGTXCs

We focused on the DMRs in promoter regions that could be directly associated with gene expression. The overlapping DMRs with a methylation difference >20% and adjusted *p*-value < 0.05 in the promoter region (1500 bp upstream and 1500 bp downstream from the putative TSS) were selected. Of these shared DMRs in NGTXCs, the DMRs exhibiting methylation changes in the same direction were selected as “overlapping” DMRs. A complete list of the overlapping DMRs in promoter regions between each NGTXC is available in Supplemental Table S8. In particular, there were 59 overlapping DMRs in the promoter region between DEHP and CA. This accounts for 31% of the 188 DMRs found in CA. However, the mode of actions (MOAs) of DEHP and CA were peroxisome proliferators and xenobiotic metabolizing enzyme inhibitors, respectively, and there was no similarity between the non-genotoxic carcinogenic mechanisms. Only three DMRs were found between OA and SS. Table 4 lists the genes harboring the overlapping DMRs in four or more NGTXCs. The 13 genes associated with overlapping DMRs in four NGTXCs were identified, of which seven were hypermethylated and six hypomethylated. Although two genes (H2-D1 and Mir705) harboring overlapping DMRs were found to be significantly differentially methylated between the transformed foci and controls across the five NGTXCs, the roles of these genes in cancer have not yet been investigated.

### 2.5. Enriched Functional Annotation and Canonical Pathway

The functions associated with genes harboring DMRs identified for each NGTXC were annotated (Table 5). For enriched function annotation, we used a list of DMRs with more than 30% changes between the samples (controls vs. the transformed foci) at a CpG site and achieving an FDR-adjusted *p*-value < 0.05. In six NGTXCs, except SA, the genes with DMRs at CpG sites were significantly enriched in cancer-related categories, including “cancer”, “cell-to-cell signaling and interaction”, and “cell death and survival”, although these were not the most enriched categories. On the other hand, the genes harboring DMRs in SA were significantly related to connective tissue development and function (*p* = 1.93 × 10^−5^). Additionally, as shown in Table 6, several canonical pathways revealed the significant enrichment of the activation pathway of the retinoid acid receptor (RAR) and retinoid X receptor (RXR) families (in six NGTXCs), gustation pathways (in six NGTXCs), as well as the salvage pathway of pyrimidine ribonucleotides (in five NGTXCs), although these pathways were not observed as top hits. Overall, the annotations represent the trends of the malignant cell transformation of cells with NGTXC treatment.

### 2.6. Network Identification

**Figure 2:**
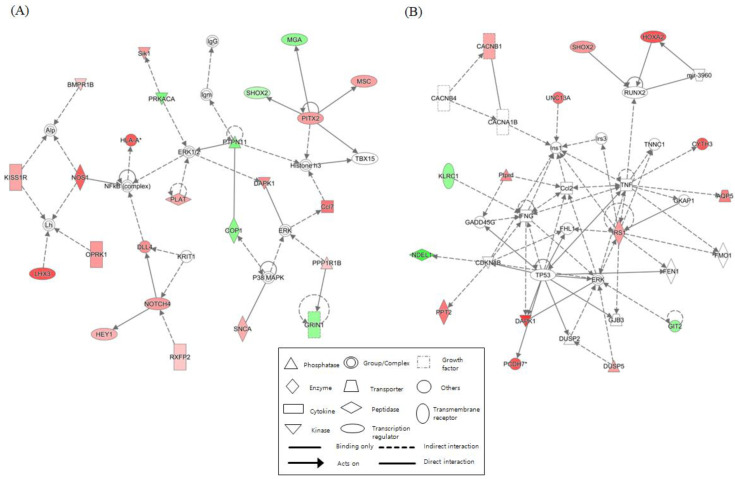
Top two networks based on ingenuity pathway analysis (IPA) showing interactions between the controls and the case. (**A**) Cell-to-cell signaling and interaction (control vs. DEHP). (**B**) Cancer, cell death and survival (control vs. OA). The lines between the genes represent known interactions (solid lines represent direct interactions; dashed lines represent indirect interactions). Each gene is displayed using shapes that represent the functional class of the gene product as indicated in the legend. The intensity of the red and green colors indicates the degree of up- and downregulation, respectively. A total of 25 networks (*p*-scores > 18) are provided in Supplementary Table S9.

We identified networks with scores over 5 for each NGTXC. Figure 2 depicts the top two highest-scoring IPA-generated networks. One network (control vs. DEHP) with a score of 32, including genes for ERK1/2, NFκB (complex), NOS1, NOTCH4, PITX2, and PTPN11, was associated with cell-to-cell signaling and interaction, gene expression, nervous system development, and function. The other network (control vs. OA) with a score of 28 included genes for Ccl2, IFNG, IRS1, RUNX2, TNF, and TP53, was associated with cancer, cell death and survival, and organismal development. Networks related to “cell death and survival”, which were considered to be linked to carcinogenesis, were identified in the other six NGTXCs, except for CA. Supplementary Table S9 features detailed information, including the molecules, score, and focus molecules, for each network.

## 3. Discussion

Genotoxic carcinogens (GTXCs) can be detected with a battery of genotoxicity tests, such as the measurement of primary DNA damage. However, NGTXCs act through a large and diverse variety of different and specific mechanisms that do not involve direct interaction with DNA [1]. Thus, in contrast to GTXCs, a panel of assays addressing particular biological endpoints will be needed for the detection of NGTXCs [27]. There is cumulative evidence that the in vitro Bhas 42 CTA is suitable for detecting NGTXCs acting via non-genotoxic mechanisms (Validation Study Report [4,5]). However, in vitro CTA approaches do not provide information on the non-genotoxic mechanisms of carcinogenicity. Recently, it has been suggested that the development of an IATA could be the mechanistic basis for the carcinogenicity of NGTXCs [27]. IATA can include a combination of methods that includes in vitro testing, QSAR, and -omics technologies.

Here, in order to identify the epigenetic modifications associated with NGTXC-induced carcinogenesis, we provided an integrated strategy consisting of (QSAR), in vitro Bhas 42 CTA, and DNA methylomes employing RRBS analysis. DNA methylation is one of the most important epigenetic modifications because aberrant methylation patterns are associated with the development diseases like cancer [45,46]. RRBS is an efficient and high-throughput technique for analyzing genome-wide methylation profiles at a single nucleotide level [25]. In addition, RRBS has been used to study several human diseases, including cancer [47], neurodegenerative diseases [48], aging [49], and immunological diseases [50]. Ten test articles selected as NGTXCs in this study were also predicted to be negative through Derek/Sarah analysis. Among the 10 NGTXCs, seven had positive results in the promotion testing of the in vitro Bhas 42 CTA. The average number of DMRs identified in the CTA-positive NGTXCs was 730–1574, with the exception of 3796 DMRs for DEHP and 288 DMRs for OA. We also discovered that, on average, 15% of the identified DMRs were located in promoter regions, which are crucial for gene expression regulation. With the exception of DEHP, the DMRs in the promoter regions showed slightly more bias toward hypermethylation than DMRs across the whole genome. Moreover, the 13 genes associated with overlapping DMRs in four NGTXCs were identified, of which seven were hypermethylated and six hypomethylated. In particular, although two genes (H2-D1 and Mir705) harboring overlapping DMRs were found to be significantly differentially methylated between transformed foci and controls across the five NGTXCs, and the roles of these genes in cancer have not yet been investigated.

To identify the major genetic events and processes occurring during malignant cell transformation by NGTXCs, we examined enriched functional annotations. The IPA analysis showed that, in six NGTXCs, except SA, the genes with DMRs at CpG sites were highly enriched in cancer-related categories, including “cancer”, “cell-to-cell signaling and interaction”, and “cell death and survival”, although these were not the most enriched categories. Overall, the annotations represent the trends of the malignant cell transformation of the cells with NGTXC treatment. Interestingly, the potential contribution of the synaptic transmission and development throughout cell transformation by NGTXCs is evident from the enrichment of genes with annotations related to “cell-to-cell signaling and interaction”. Although immunological synapses have been described previously in the context of the immune escape of glioma [51], their role in cell transformation has not been determined as of yet.

Several canonical pathways were established to have a significant enrichment of the activation pathway of the RAR and RXR families (in six NGTXCs), gustation pathways (in six NGTXCs), as well as the salvage pathway of pyrimidine ribonucleotides (in five NGTXCs), though these pathways were not observed as top hits. With this, the cell growth and differentiation of malignant cells by retinoids has been reported to be regulated by RARs and RXRs [52]. In addition, our results are in line with previous reports indicating that gustation pathways and the salvage pathway of pyrimidine ribonucleotides were identified via IPA in cancer cells [53,54]. IPA also determined the functional networks relevant to NGTXC-induced cell transformation. The most significant network was defined as “cell-to-cell signaling and interaction” (with a score of 32), where genes including ERK1/2, NFκB (complex), NOS1, NOTCH4, PITX2, and PTPN11 are clustered. The second top-ranked network was “cell death and survival” (with a score of 28).

Taken together, we posit that the genes with DMRs at CpG sites in the transformed foci are linked to malignant cell transformation that the cells undergo during NGTXC treatment. Thus, the framework in this study demonstrated the possibility of using a combination of several tools to take advantage of an integrated approach. However, the functional annotations and canonical pathways identified through our IPA analysis did not exhibit a robust correlation with the modes of action previously reported for each NGTXC. Therefore, further study will be required for the development of a more efficient integrated approach that includes a methylation profile analysis.

## 4. Materials and Methods

### 4.1. Materials

The 10 NGTXCs used for the Bhas 42 CTA in this study were as follows: methapyrilene hydrochloride (MH) (CAS # 135-23-9), D-limonene (CAS # 5989-27-5), bis(2-ethylhexyl) phthalate (DEHP) (CAS # 117-81-7), 2,3,7,8-tetrachlorodibenzo-p-dioxin (TCDD) (CAS # 1746-01-6), okadaic acid (OA) (CAS # 78111-17-8), cholic acid (CA) (CAS # 81-25-4), diethanolamine (DEA) (CAS # 111-42-2), rosuvastatin (CAS # 287714-41-4), melamine (CAS # 108-78-1), and sodium saccharin (SA) (CAS # 128-44-9). All were obtained from Sigma-Aldrich (St. Louis, MO, USA). Dulbecco’s modified Eagle’s medium/F12 (DMEM/F12), minimum essential medium (MEM), and fetal bovine serum (FBS) were purchased from Thermo Fisher Scientific (Carlsbad, CA, USA). Crystal violet (CV) and Giemsa staining solution were purchased from Sigma-Aldrich. In addition, 12-O-tetradecanoylphorbol 13-acetate (TPA, CAS # 16561-29-8) was included as a positive control for the promotion assay. Test articles were dissolved in sterile distilled water or DMSO. The final concentration of distilled water was 1% in the culture medium, and that of DMSO was 0.1%.

### 4.2. Cell Lines and Cell Culture

Bhas 42 cells, which were established from the BALB/c 3T3 cells through transfection with the v-Ha-*ras* gene [55], were obtained from the Japan Health Sciences Foundation Health Science Research Resources Bank (Osaka, Japan). As previously described [24], the cells were grown in MEM supplemented with 10% FBS (M10F). Only 60–70% of the confluent cells were sub-cultured. The cells were cultured in a humidified incubator under 5% CO_2_ and 95% O_2_. Three days prior to treatment with test articles, Bhas 42 cells were maintained in DMEM/F12 supplemented with 5% FBS (DF5F).

### 4.3. Cell Growth Assay

The cell growth assays were performed prior to the transformation assays to determine the appropriate treatment concentrations of the test articles, as well as concurrently with every transformation assay to estimate the effect of the test articles on cell growth and survival. The cells were seeded, treated with a test article, and cultured until day 7. Three wells were prepared for each treatment group. On day 7, the cells were fixed with 10% formalin and stained with a 0.1% CV solution in 5% ethanol. CV was extracted from the stained cells with a solution containing 0.02 mol/L hydrochloric acid in 50% ethanol. The optical density of the extract was measured at a wavelength between 540 and 570 nm.

### 4.4. In Vitro Bhas 42 CTA for the Promotion Test

The in vitro transformation assays were conducted according to the procedures reported by Sakai et al. [7] with slight modifications. The Bhas 42 cells were seeded into each well of 6-well microplates at 2 mL volumes (7000 cells/well, day 0). Six wells were prepared for each treatment group. The cells were cultured for four days without medium exchange. On day 4, day 7, and day 11, the culture medium was replaced with fresh medium containing a test article or vehicle alone, and the treatment in the promotion phase was continued until day 14. The media were not changed during the last 7 days of culture. The cells were then fixed with methanol and stained with Giemsa solution.

### 4.5. Isolation of Transformed Foci

For the methylation profiling analysis, the foci that formed in the culture dish were cloned with cloning cylinders (6 mm internal diameter, 8 mm external diameter, 8 mm length). The foci were inspected, and the desired foci were circled. Growth medium was removed from the dish and cells washed with PBS. Then, a sterile cloning cylinder with one edge coated with silicone grease was placed around the selected focus, and 40 μL of trypsin was added to the cloning cylinder well. Cell lines were established from each focus, which were placed in 6-well plates containing M10F. After cellular growth for 10 to 15 days with frequent changes of medium, the genomic DNA from one established cell line was analyzed for methylation with RRBS.

### 4.6. Genomic DNA Extraction and Reduced-Representation Bisulfite Sequencing (RRBS)

For the whole-genome DNA, RRBS was performed by C&K Genomics, Inc. (Seoul, Korea) Briefly, genomic DNA was quantified in a Victor2 spectrophotometer (PerkinElmer, Boston, MA, USA) using the Quant-iT™ PiboGreen™ RNA Assay Kit (Invitrogen, Carlsbad, CA, USA) according to the manufacturer’s protocol. To construct the Msp1 and Apek1 digested RRBS library, 500 ng of genomic DNA was incubated at 37 °C for 24 h. The digested products were purified with a MinElute PCR Purification Kit (Qiagen, Valencia, SC, USA). After purification, the digested products were blunt-ended, and then the dA was added, followed by methylated-adapter ligation. A range of 160–420 adapter-ligated fractions was excised from a 2% agarose gel. Bisulfite conversion was conducted using a ZYMO EZ DNA Methylation-Gold Kit™ (ZYMO research, Irvine, CA, USA) following the manufacturer’s instructions. The final libraries were generated by PCR amplification using PfuTurbo Cx Hotstart DNA Polymerase (Agilent Technologies, Santa Clara, CA, USA). RRBS libraries were evaluated by an Agilent 2100 Bioanalyzer (Agilent Technologies, Palo Alto, CA, USA). Libraries were quantified by qPCR using the CFX96 Real-Time System (Bio-Rad, Hercules, CA, USA). After normalization, the sequencing of the prepared library was conducted on the Nextseq system (Illumina, San Diego, CA, USA) with 75-bp paired-end reads.

### 4.7. RRBS Data Analysis

RRBS data were preprocessed by the following steps. Briefly, low-quality adapter sequences in the raw reads were trimmed using TrimGalore (version 0.6.5) with Cutadapt (version 1.18) [56]. Quality checks of the clean data were conducted with FastQC (version 0.11.5), then high-quality reads were mapped to the reference genome (Mouse genome assembly GRCm38.p6) using Bismark (version 0.22.3) with bowtie2 (version 2.3.5) [57]. Data quality was shown in Supplementary Table S10. During the NGTXC-induced cell transformation, the DMRs were identified using DMRcaller (version 1.18.0), which uses the Bismark output data as input [58]. We considered 100-bp tilling bins for computing the differentially methylated cytosines, and performed a score test between the methylated and total reads in a bin for the samples (untransformed cells vs. transformed foci). We selected the bins where the FDR-adjusted *p*-value was less than 0.05 and the difference in level of methylation was at least 20% in the CG context, containing at least four cytosines (with at least four reads per cytosine). The identified DMRs were annotated using ChipSeeker [59] with gene annotation information from the R package TxDb.Mmusculus.UCSC.mm10.knownGene and org.Mm.eg.db [60]. Promoters were defined as the DMRs within 1500 bp upstream and 1500 bp downstream from the TSS.

### 4.8. Molecular Network, Pathway, and Functional Analysis

Enriched functional annotation, gene network identification, and canonical pathway analysis (generalized pathways that represent the common properties of a particular signaling module or pathway) were performed using Ingenuity Pathway Analysis software (Ingenuity Systems, Redwood City, USA). We utilized a list of DMRs with more than 30% changes between the samples (controls vs. transformed foci) at a CpG site and achieving an FDR-adjusted *p*-value < 0.05. The networks were identified using the scores calculated for each network according to the fit of the network to the set of the focus genes of the ingenuity analysis. The significance of the biofunctions and canonical pathways were tested by *p*-value. In addition, the canonical pathways were ranked by the ratio (number of genes from the input DMR data that maps to the pathway divided by the total number of molecules that exist in the canonical pathway).

## Figures and Tables

**Table 1:** Published genotoxicity and carcinogenicity results for the test articles.

Test Articles	Genotoxicity	Carcinogenicity	ICH M7 Prediction (Derek/Sarah)	OECD QSAR Toolbox Prediction
Methapyrilene hydrochloride (MH)	Equivocal in Ames test, Negative in in vitro CA and SCE, in vivo CA, SCE and MN	Potent hepatocarcinogen in rats	-/-	No alert found
D-limonene	Negative in Ames test, in vivo comet, in vitro CA	Male rat kidney tumors	-/-	No alert found
bis(2-ethylhexyl) phthalate (DEHP)	Negative in Ames test, in vivo MN, in vitro MN, MLA	IARC Group 2B	-/-	Structural alert for nongenotoxic carcinogenicity
TCDD	Negative in Ames test, in vitro MLA, CA, SCE, in vivo CA	IARC Group 1	-/-	Structural alert for nongenotoxic carcinogenicity
Okadaic acid (OA)	Negative in Ames test, in vitro CHO/HGPRT, but mutagenic to CHL cells	Tumor promoter on mouse skin	-/-	No alert found
Cholic acid (CA)	Equivocal in Ames test, non-significant mutagenic activity in a battery of in vitro genotoxicity tests	Colon cancer promoter	-/-	No alert found
Diethanolamine (DEA)	Negative in Ames test, in vivo MN, in vitro CA	IARC Group 2B	-/-	No alert found
Rosuvastatin	Negative in Ames test, in vivo MN, in vitro CA, MLA	Positive response in mice and rats	-/-	Structural alert for nongenotoxic carcinogenicity
Melamine	Negative in Ame test, in vivo MN, in vitro CA	Bladder carcinoma	-/-	Structural alert for genotoxic carcinogenicity
Sodium saccharin (SA)	Negative in Ames test, in vivo CA and comet, in vitro MLA	Rat and mouse bladder tumors	-/equivocal	Structural alert for nongenotoxic carcinogenicity

Abbreviations: Ames, reverse bacterial mutation assay in *Salmonella typhimurium*; in vitro CA, in vitro chromosomal aberration assay; in vivo MN, micronucleus test; SCE, sister chromatid exchange; MLA, mouse lymphoma assay; CHO, Chinese hamster ovary; CHL, Chinese hamster lung; HGPRT, hypoxanthine guanine phosphoribosyltransferase; IARC, International Agency for Research on Cancer; -, negative response. References: D-limonene, melamine, diethanolamine. DEHP, rosuvastatin, sodium saccharin [28]; ethapyrilene hydrochloride [32]; TCDD [33]; Okadaic acid [34].

**Table 2:** Mode of actions of the test articles.

Test Articles	Mode of Actions	Reference
Methapyrilene hydrochloride	The dysregulation of hepatic iron metabolism	[35]
D-limonene	α2 μ-globulin nephropathy	[36]
Bis(2-ethylhexyl) phthalate (DEHP)	Peroxisome proliferators	[37]
TCDD	Receptor binding	[38]
Okadaic acid	Inhibitors of protein serine/threonine phosphatases	[39]
Cholic acid	Inhibition of xenobiotic metabolizing enzyme	[40]
Diethanolamine	Choline deficiency	[41]
Rosuvastatin	Inhibitor of 3-hydroxy-3-methylglutaryl-coenzyme A (HMG-CoA) reductase	[42]
Melamine	Calculus formation	[43]
Sodium saccharin	Calculus formation	[44]

**Table 3:** Bhas 42 cell transformation assay (Promotion test).

Conc.	% Viability	Foci/Well	Conc.	% Viability	Foci/Well
Sodium saccharin (128-44-9)—Positive			Methapyrilene HCl (135-23-9)—Positive		
0 (μg/mL)	100	3.5 ± 1.38 ^(a)^	0 (μg/mL)	100	7.2 ± 0.75
500	107	9.3 ± 1.97 **^,(b)^	3	107	14.5 ± 2.59 **
750	113	8.8 ± 1.94 **	3.5	110	16.0 ± 1.26 **
1000	93	9.3 ± 1.03 **	4	101	13.8 ± 1.60 **
1250	106	9.7 ± 1.21 **	4.5	100	15.0 ± 2.00 **
1500	94	8.2 ± 1.94 **	5	96	14.5 ± 2.43 **
1750	101	7.3 ± 2.16 **	5.5	99	10.5 ± 3.27
DEHP (117-81-7)—Positive			D-limonene (5989-27-5)—Negative		
0 (μg/mL)	100	11.0 ± 1.79	0 (μg/mL)	100	12.7 ± 3.01
2.5	93	13.8 ± 2.48	12.5	103	14.7 ± 3.72
5	92	19.5 ± 2.17 **	15	103	12.2 ± 3.19
10	91	20.5 ± 4.76 **	17.5	102	14.0 ± 2.00
25	95	18.5 ± 3.73 **	20	101	12.8 ± 3.60
50	93	18.5 ± 1.64 **	22.5	83	12.3 ± 2.07
100	90	13.0 ± 3.52	25	78	9.8 ± 2.14
Melamine (108-78-1)—Negative			Rosuvastatin (287714-41-4)—Negative		
0 (μM)	100	7.5 ± 1.76	0 (μg/mL)	100	8.0 ± 0.89
0.25	92	7.8 ± 2.23	0.1	103	8.8 ± 1.83
0.5	96	8.2 ± 2.48	0.25	100	7.8 ± 1.83
1	95	9.0 ± 2.68	0.5	102	8.7 ± 1.51
2.5	98	9.7 ± 3.08	0.75	117	8.2 ± 1.94
5	96	10.8 ± 2.23	1	125	9.8 ± 3.13
10	103	9.3 ± 3.20	2	116	8.7 ± 1.63
Diethanolamine (111-42-2)—Positive			Okadaic acid (78111-17-8)—Weak positive		
0 (μg/mL)	100	7.8 ± 1.94	0 (ng/mL)	100	8.8 ± 1.17
10	99	14.0 ± 3.22 *	0.25	100	11.7 ± 2.25
25	117	16.7 ± 2.66 **	0.5	109	13.0 ± 3.35 *
50	114	12.8 ± 3.87	0.75	97	11.2 ± 3.49
75	110	17.5 ± 2.07 **	1	102	12.8 ± 2.14 *
100	88	16.8 ± 3.66 **	2	118	6.2 ± 0.75
150	112	17.8 ± 4.71 **	3	117	5.0 ± 1.41
TCDD (1746-01-6)—Positive			Cholic acid (81-25-4)—Positive		
0 (nM)	100	11.3 ± 1.37	0 (μg/mL)	100	5.0 ± 2.28
10	77	20.0 ± 3.35 **	10	124	8.8 ± 2.48
25	86	19.3 ± 3.14 **	25	119	9.0 ± 1.79 *
50	81	21.2 ± 0.75 **	50	107	9.3 ± 1.97 *
75	85	18.8 ± 3.43 **	100	122	9.0 ± 3.22 *
100	89	19.7 ± 2.25 **	250	103	10.0 ± 2.68 **
200	79	17.3 ± 1.86 **	500	131	11.8 ± 2.99 **

^(a)^ Values represent the mean SD of six wells per group. ^(b)^ ** *p* < 0.01 and * *p* < 0.05 as compared with vehicle control, as determined by Dunnett’s *t* test.

**Table 4:** Genes harboring the overlapping DMRs.

Associated Gene	Description	DMR Position	DMR Distance from TSS ^(1)^	DMCs Number	Methylation	NGTXC
**Genes harboring overlapping DMRs in 4 NGTXCs, -induced transformed foci**						
Asic1	Acid-sensing (proton-gated) ion channel 1	chr15: 99,691,202–99,691,300	−933	8	hypomethylated	DEHP/OA/TCDD/MH
Gm14169	Predicted gene 14169	chr2: 156,613,303–156,613,401	21	18	hypermethylated	CA/DEHP/OA/TCDD
Gm7337	DAZ interacting protein 1 pseudogene	chr5: 87,851,102–87,851,200	584	10	hypomethylated	CA/DEHP/OA/MH
Gpsm2	G-protein signaling modulator 2	chr3: 108,721,702–108,721,900	216	26	hypomethylated	CA/DEHP/OA/TCDD
Irs1	Insulin receptor substrate 1	chr1: 82,290,404–82,290,502	914	18	hypermethylated	OA/TCDD/DEA/SS
Mpv17	MpV17 mitochondrial inner membrane protein	ch5: 31,154,402–31,154,500	−151	8	hypermethylated	CA/DEHP/OA/TCDD
Nexn	Nexilin	chr3: 152,265,802–152,265,900	−7	22	hypomethylated	DEHP/DEA/MH/SS
Nrip2	Nuclear receptor interacting protein 2	chr6: 128,401,302–128,401,400	1009	2	hypomethylated	CA/DEHP/TCDD/SS
Nsun7	NOL1/NOP2/Sun domain family, member 7	chr5: 66,261,102–66,261,200	790	10	hypomethylated	CA/TCDD/DEA/SS
Pcdh7	Protocadherin 7	chr5: 57,720,202–57,720,300	79	14	hypermethylated	CA/OA/DEA/MH
Pou6f1	POU domain, class 6, transcription factor 1	chr15: 100,599,102–100,599,200	664	6	hypermethylated	CA/DEHP/TCDD/SS
Unc13a	Unc-13 homolog A	chr8: 71,668,402–71,668,500	−320	12	hypermethylated	CA/DEHP/OA/TCDD
Zfp882	Zinc finger protein 882	chr8: 71,908,855–71,908,954	0	14	hypermethylated	TCDD/DEA/MH/SS
**Genes harboring overlapping DMRs in 5 NGTXCs-induced transformed foci**						
H2-D1	Histocompatibility 2, D region locus 1	chr17: 35,263,302–35,263,400	206	23	hypomethylated	CA/DEHP/DEA/MH/SS
Mir705	MicroRNA 705	chr6: 85,337,102–85,337,200	−729	6	hypomethylated	DEHP/OA/DEA/MH/SS

^(1)^ TSS, transcription start site (1500 bp downstream and 1500 bp upstream from TSS)—> Promoter. Abbreviation: DMCs, differentially methylated cytosines

**Table 5:** Top 3 and significant enriched functional annotations of the differentially methylated regions between the control and the NGTXC-treated group (*p*-value < 0.05 and proportion of methylation difference >0.3).

NGTXC	Categories	Disease or Functions Annotation	*p*-Value	Number of Molecules
Cholic acid	Cellular assembly and organization	Release of vesicles	0.00605	2
	Lipid metabolism	Concentration of malonyl-coenzyme A	0.00913	1
	Embryonic development	Attachment of chorioallantoic membrane	0.00913	1
	Cancer, cellular growth and proliferation	Proliferation of mammary tumor cells	0.0182	1
DEHP	Behavior	Learning	7.93 × 10^−6^	25
	Cell-to-cell signaling and interaction	Uptake of neurotransmitter	1.85 × 10^−5^	5
	Neurological Disease	Cognitive impairment	0.00009	16
	Cell death and survival	Apoptosis of tumor cell lines	0.0053	13
Okadaic aicd	Gastrointestinal disease	Meteorism	0.000468	2
	Cell-to-cell signaling and interaction	Developmental process of synapse	0.000555	4
	Respiratory disease	Abnormal secretion of pulmonary surfactant	0.00135	2
TCDD	Cellular movement	Cellular infiltration by macrophages	7.68 × 10^−6^	9
	Behavior	Long-term recognition memory	5.16 × 10^−5^	4
	Cardiovascular system development and function	Systolic pressure	0.00014	7
	cancer	Cell transformation	0.00125	9
	Cell death and survival	Apoptosis	0.00531	17
Diethanol-amine	Embryonic development	Formation of visceral endoderm	0.000195	2
	Cell cycle	Aneuploidy of embryonic cell lines	0.000291	2
	Cell cycle	Aneuploidy of fibroblast cell lines	0.000407	2
	Cell-to-cell signaling and interaction	Developmental process of synapse	0.00116	4
	Cancer	Hyperplasia of urothelium	0.00446	1
Methapyrilene HCl	Connective tissue development and function	Osteogenesis	0.00316	2
	Cell-to-cell signaling and interaction	Synaptic transmission	0.00793	6
	Reproductive system development and function	Gestation	0.00814	3
Sodium saccharin	Connective tissue development and function	Thickness of bone	1.93 × 10^−5^	7
	Skeletal and muscular system development and function	Mineralization of bone	3.44 × 10^−5^	8
	Connective tissue disorders	Synostosis of cranium	6.38 × 10^−5^	2
	Behavior	Social behavior	9.72 × 10^−5^	6

**Table 6:** Top 3 and the significant canonical pathways of the differentially methylated regions between the control and the NGTXC-treated group (*p*-value < 0.05 and proportion of methylation difference >0.3).

NGTXC	Ingenuity Canonical Pathways	−log (*p*-value)	Ratio	Molecules
Cholic acid	Notch signaling	2.37	0.0833	CNTN1, DTX1, LFNG
	Axonal guidance signaling	1.97	0.0214	ADAM19, ADAMTS1, GNG2, MYL12A, PRKAR1B, SEMA4A, SEMA4G, SEMA6C, SRGAP1, SRGAP3
	Thyroid hormone biosynthesis	1.57	0.333	IYD
	RAR activation	1.02	0.0211	Akr1b10, NRIP2, PRKAR1B, SMARCD3
	Gustation pathway	0.863	0.0214	GNG2, PDE1B, PRKAR1B
	Salvage pathways of pyrimidine ribonucleotides	0.695	0.022	DAPK1, GRK4
DEHP	Dopamine-DARPP32 feedback in cAMP Signaling	3.54	0.0692	GNAI1, GRIN1, GRIN3B, KCNJ12, NOS1, PPP1R1B, PPP2R5A, PRKACA, PRKAR1B, PRKCZ, PRKG2
	Notch signaling	2.3	0.111	DLL4, DTX1, HEY1, NOTCH4
	nNOS signaling in neurons	1.95	0.0889	GRIN1, GRIN3B, NOS1, PRKCZ
	Gustation pathway	1.25	0.0429	ASIC1, GNG2, P2RY12, PDE6B, PRKACA, PRKAR1B
	Salvage pathways of pyrimidine ribonucleotides	0.585	0.033	AK1, DAPK1, GRK4
Okadaic aicd	Glutamate degradation II	1.96	0.333	GOT2
	L-Serine degradation	1.96	0.333	SDS
	Aspartate biosynthesis	1.96	0.333	GOT2
	RAR activation	1.49	0.0158	Akr1b10, MAP2K5, NSD1
	Salvage pathways of pyrimidine ribonucleotides	1.36	0.022	DAPK1, GRK4
	Gustation pathway	1.03	0.0143	ASIC1, CACNB1
TCDD	Pyridoxal 5′-phosphate salvage pathway	2.48	0.0625	GRK4, MAP2K6, PDXK, PKN1
	PPARα/RXRα activation	2.1	0.0331	APOA1, GOT2, IRS1, MAP2K6, PPARA, PRKAR1A
	Apelin liver signaling pathway	1.6	0.0769	APLNR, IRS1
	Salvage pathways of pyrimidine ribonucleotides	1.25	0.033	GRK4, MAP2K6, PKN1
	Gustation pathway	0.824	0.0214	ASIC1, CACNA1D, PRKAR1A
Diethanolamine	Regulation of the epithelial–mesenchymal Transition pathway	1.99	0.0211	CLDN3, FGF19, FGFR2, NOTCH1
	Glutamate degradation II	1.88	0.333	GOT2
	Aspartate Biosynthesis	1.88	0.333	GOT2
	Cancer drug resistance by drug efflux	1.56	0.0345	miR-133, PTEN
	Gustation pathway	0.889	0.0143	CACNB1, PDE8B
	PPARα/RXRα activation	0.714	0.011	GOT2, IRS1
Methapyrilene HCl	γ-glutamyl cycle	4.14	0.3	GGCT, GGT6, GGT7
	Leukotriene biosynthesis	2.34	0.167	GGT6, GGT7
	Valine degradation I	1.99	0.111	ABAT, SDS
	Autophagy	1.05	0.0345	CTSF, ULK1
	Phagosome maturation	0.921	0.0214	CTSF, HLA-A, TCIRG1
	VDR/RXR activation	0.839	0.0256	BGLAP, IGFBP5
	Salvage pathways of pyrimidine Ribonucleotides	0.264	0.011	CDK5
Sodium saccharin	Gustation pathway	3.03	0.0429	P2RX7, P2RY12, PDE1B, PDE8B, PDE9A, PRKACA
	tRNA splicing	2.37	0.0732	PDE1B, PDE8B, PDE9A
	G-protein coupled receptor signaling	1.68	0.0225	P2RY12, PDE1B, PDE8B, PDE9A, PRKACA, PRKCB
	VDR/RXR activation	1.61	0.0385	PRKCB, RUNX2, SPP1
	Phagosome maturation	1.58	0.0286	ATP6V1G3, HLA-A, LPO, TCIRG1

## Supplementary Materials

Supplementary materials can be found at https://www.mdpi.com/1422-0067/21/15/5387/s1. Table S1. List of DMRs associated with cholic acid (CA); Table S2. List of DMRs associated with bis(2-ethylhexyl) phthalate (DEHP); Table S3. List of DMRs associated with okadaic acid (OA); Table S4. List of DMRs associated with 2,3,7,8-tetrachlorodibenzo-p-dioxin (TCDD); Table S5. List of DMRs associated with diethanolamine (DEA); Table S6. List of DMRs associated with methapyrilene hydrochloride (MH); Table S7. List of DMRs associated with sodium saccharin (SS). Table S8; List of overlapping DMRs in a promoter region between each NGTXC. Table S9; Networks with a score with scores over 5 in each NGTXC. Table S10; Summary of the data quality of RRBS performance. Figure S1. Association between DMRs and cell transformation.

## Abbreviations

CTA	In vitro cell transformation
NGTXC	Non-genotoxic carcinogen
DMR	Differentially methylated region
RRBS	Reduced-representation bisulfate sequencing
IATA	Integrated approach to testing and assessment
IPA	Ingenuity pathway analysis
QSAR	Quantitative structure–activity relationship
MH	Methapyrilene hydrochloride
DEHP	Bis(2-ethylhexyl) phthalate
TCDD	2,3,7,8-tetrachlorodibenzo-p-dioxin
OA	Okadaic acid
CA	Cholic acid
DEA	Diethanolamine
SA	Sodium saccharin
