# Supplementary material for: Predicting Carcinogenic Mechanisms of Non-Genotoxic Carcinogens via Combined Analysis of Global DNA Methylation and In Vitro Cell Transformation
Source: Int J Mol Sci. 2020 Jul 29;21(15):5387. doi: 10.3390/ijms21155387 (PMC7432388; doi:10.3390/ijms21155387)
Supplement: Supplementary file 1 [file ijms-21-05387-s001.zip › Suppl Figure S1.pptx]

## Slide 1
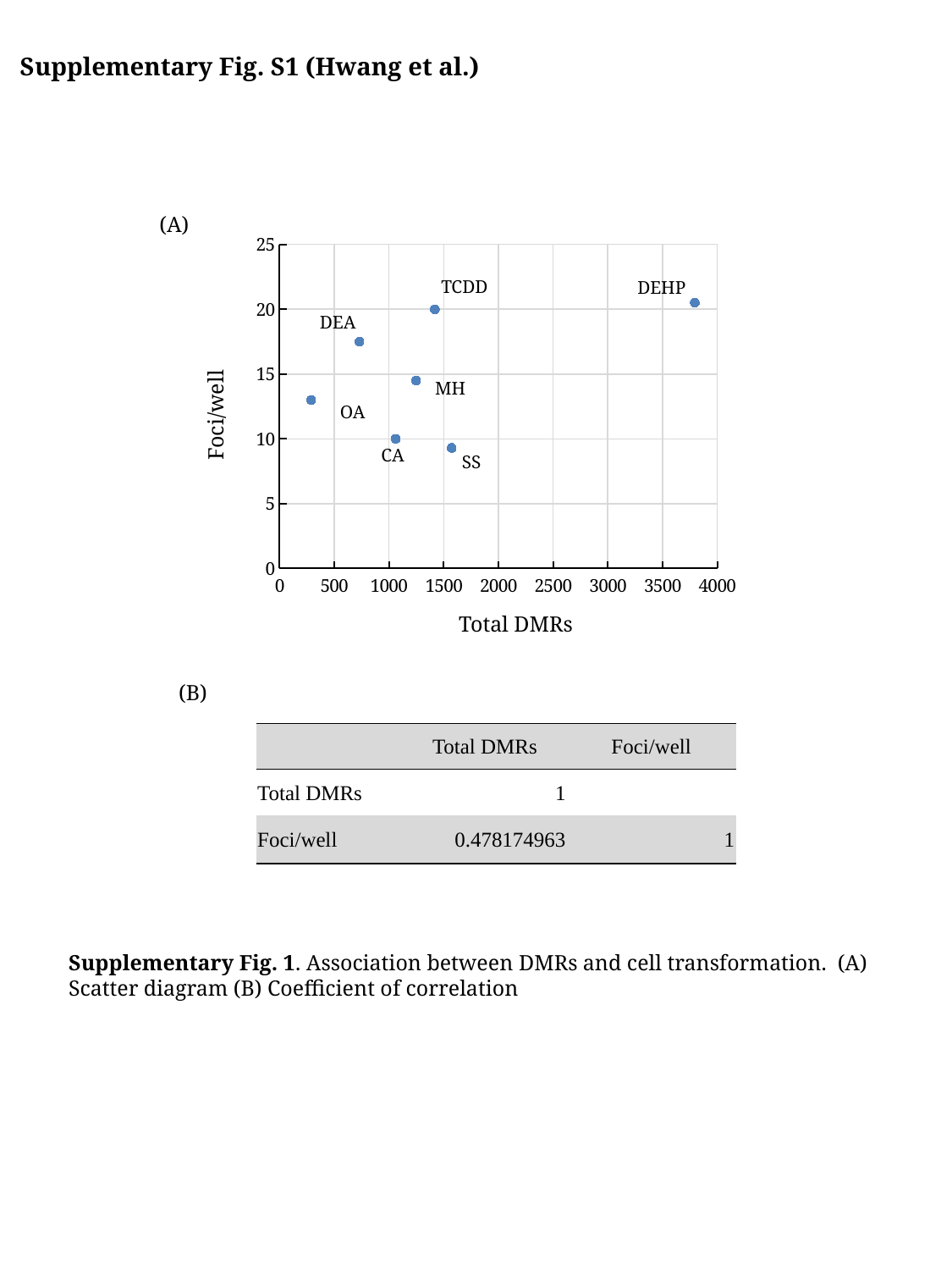

Supplementary Fig. S1 (Hwang et al.)
(A)
### Chart
| Category | In vitro CTA (foci/well) |
|---|---|TCDD
DEHP
DEA
MH
OA
Foci/well
CA
SS
Total DMRs
(B)
| | Total DMRs | Foci/well |
| --- | --- | --- |
| Total DMRs | 1 | |
| Foci/well | 0.478174963 | 1 |
Supplementary Fig. 1. Association between DMRs and cell transformation. (A) Scatter diagram (B) Coefficient of correlation
